# The serum angiotensin converting enzyme and lysozyme levels in patients with ocular involvement of autoimmune and infectious diseases

**DOI:** 10.1186/s12886-016-0194-4

**Published:** 2016-02-16

**Authors:** Ozlem Sahin, Alireza Ziaei, Eda Karaismailoğlu, Nusret Taheri

**Affiliations:** Department of Ophthalmology/Uveitis, Dunya Goz Hospital Ltd., Ankara, Turkey; Department of Ophthalmology, Boston University School of Medicine, Boston, MA USA; Department of Biostatistics, Hacettepe University Medical Faculty, Ankara, Turkey; Department of Biochemistry, Middle East Technical University, Health Sciences, Ankara, Turkey

**Keywords:** Angiotensin converting enzyme, Lysozyme, Ankylosing spondylitis, Behcet’s disease, Sarcoidosis, Syphilis, Tuberculosis, Ocular inflammation

## Abstract

**Background:**

Increased serum levels of angiotensin converting enzyme and lysozyme are considered as inflammatory markers for diagnosis of sarcoidosis which is an autoimmune inflammatory disease. The purpose of this study is to evaluate the significance of differences in serum angiotensin converting enzyme and lysozyme levels of patients with ocular involvement of other autoimmune inflammatory and infectious diseases.

**Methods:**

This is a prospective study involving patients with ankylosing spondylitis, behcet’s disease, presumed sarcoidosis, presumed latent tuberculosis, presumed latent syphilis, and control group. The serum levels of angiotensin converting enzyme and lysozyme were analyzed by enzyme-linked immunosorbent assay. Bonnferoni analysis was used to assess pairwise comparisons between the groups.

**Results:**

There was a significant increase in serum angiotensin converting enzyme level in patients with presumed sarcoidosis compared to ankylosing spondylitis (*p* = 0.0001), behcet’s disease (*p* = 0.0001), presumed latent tuberculosis (*p* = 0.0001), presumed latent syphilis (*p* = 0.0001), and control group (*p* = 0.0001). The increase in serum lysozyme level was significant for patients with presumed sarcoidosis with respect to ankylosing spondylitis (*p* = 0.0001), behcet’s disease, (*p* = 0.0001) presumed latent tuberculosis (*p* = 0.001), presumed latent syphilis (*p* = 0.033), and control group (*p* = 0.0001).

**Conclusion:**

Elevated serum angiotensin converting enzyme levels are significant for patients with presumed sarcoidosis compared to ocular involvement of other autoimmune diseases such as behcet’s disease and ankylosing spondylitis, and ocular involvement of infectious diseases such as presumed latent tuberculosis and presumed latent syphilis. However, elevated serum lysozyme level might be also detected in ocular involvement of infectious diseases such as presumed latent tuberculosis and presumed latent syphilis.

**Trial registration:**

Trial Registration number: NCT02627209. Date of registration: 12/09/2015.

## Background

The renin angiotensin system (RAS) is an important hormonal system which promotes inflammation through the axis of angiotensin converting enzyme (ACE)-angiotensin peptides-distinct receptor subtypes [1]. Hyperactivation of the RAS system has been disclosed to involve in inflammatory responses of the eye [2, 3]. Increased serum ACE activity has been reported especially in uveitis associated with sarcoidosis, and also infectious uveitis such as recurrent toxoplasmic and toxocaral iridocyclitis and choroioretinitis [4, 5]. Lysozyme is an enzyme that hydrolyses glycosidic bonds, and it is revealed to degrade the peptidoglycans in the bacterial cell wall [6]. Muramidase activity of lysozyme has been shown to limit the inflammation caused by rapidly degrading peptidoglycans at the site of infection [7]. Elevated levels of serum lysozyme have been reported in granulomatous uveitis especially sarcoidosis and tuberculosis [8]. The purpose of this study is to evaluate the significance of differences in serum ACE and lysozyme levels of patients with ocular involvement of autoimmune diseases such as HLAB27^+^ ankylosing spondylitis (AS), HLAB51^+^ behcet’s disease (BD) and presumed sarcoidosis and ocular involvement of infectious diseases such as QuantiFERON(®)-TB Gold^+^ presumed latent tuberculosis (TB) and presumed latent syphilis compared to control group by using pairwise comparisons between the groups.

## Methods

### Study patients

This is a prospective study involving 76 patients with AS, 72 patients with BD, 31 patients with presumed sarcoidosis, and 68 patients with presumed latent TB, 11 patients with presumed latent syphilis, and 22 control subjects having refractive errors only. Institutional review board/ethics committee approval was obtained from the Dunya Goz Hospital Institutional Board (DGH-070) Ankara, Turkey. This study adhered to the tenets of the Declaration of Helsinki. The written informed consent was obtained for all individuals who enrolled in this study. The patients had ocular manifestations including acute or chronic granulomatous or non-granulomatous iritis or iridocyclitis, intermediate, posterior or panuveitis, retinitis, retinal vasculitis, choroiditis and papillitis. The uveitis was not always granulomatous inside the the group of presumed ocular sarcoidosis. The international criteria for the diagnosis of presumed ocular sarcoidosis and international study criteria for BD were used [9, 10]. Inclusion criteria for all patients involve the presence of ocular signs associated with a positive test for HLAB27 for patients with AS, a positive test for HLAB51 for the patients with BD, a negative tuberculin skin test in BCG-vaccinated patients, positive findings on chest x-ray or chest CT or abnormal results on whole body Gallium 67 scintigraphy for the patients with presumed sarcoidosis, a positive test for QuantiFERON(®)-TB Gold In-Tube (Cellestis limited, Melbourne, Australia) and a positive tuberculin skin test for the patients with presumed latent TB. Presumed latent syphilis was diagnosed on the basis of negative serum venereal disease research laboratory test (VDRL) and rapid plasma reagin test, (RPR) positive serum fluorescent treponemal antibody absorption (FTAABS) and microhemagglutination assay for Treponema pallidum, (MHA-TP), negative cerebrospinal fluid (CSF) FTAABS, and improvement in level of ocular inflammation after treatment with specific therapy for syphilis [11]. The difference in the levels of ACE in granulomatous and a non-granulomatous groups were not compared. The connection of increased serum lysozyme levels between systemic diseases such as sarcoidosis was not studied. The exclusion criteria for the patient and control groups were using ACE inhibitors, systemic steroids, immunosuppressive or immunomodulatory therapies.

### Blood sampling

Blood samples were collected from the patients by using a standard aseptic technique. Native blood was incubated for 60 min at room temperature; serum fractions (separated by centrifugation at 1500 *g* for 15 min) were stored at −20 °C.

### Serum ACE activity measurement using spectrophotometric assay

Serum ACE activity was measured as described by Beneteau et al [12]. ACE activity was determined with an artificial substrate (FAPGG, (*N*-[3-(2-furyl) acryloyl]-L-phenylalanyl-glycylglycine; Sigma-Aldrich) in a reaction mixture containing 25 mM HEPES (*N*-2-hydroxyethylpiperazine-*N*-2-ethanesulfonic acid), 0.5 mM FAPGG, 300 mM NaCl, and the desired dilution of serum at pH 8.2. Measurements were performed in 96-well plates (Greiner-Bio One) at 37 °C. Changes in optical density (340 nm) were measured at 5-min intervals for at least 90 min with a plate reader (NovoStar plate reader; BMG Labtech). Optical density values were plotted as a function of reaction time and fitted by linear regression (GraphPad Prism 5.0). The fit and the data were accepted when *r*^*2*^ was >0.90. ACE activity was calculated with the equation:

$$ activity=\left(S/k\right)D $$

where S is the rate of observed decrease in optical density (1/min), k is the change in optical density upon the complete cleavage of 1 μmol of FAPGG, and D is the dilution of the serum. ACE activity is given in units where 1 U is equivalent to the cleavage of 1 μmol of FAPGG in 1 min. The reference range for serum ACE level was between 8 and 52 U/L for adults, and between 13 and 100 U/L for children less than 18 years of age.

### Serum lysozyme activity measurement using radial immunodiffusion

Serum lysozyme activity was measured by using radial immunodiffusion as described by Mancini G. et al. [13]. Human ‘NL’ Nanorid plate, No GT073.3, (Binding Site Ltd., Birmingham, UK) and lysozyme - NL 2.1–21* GT073.3 NANORID™ kits (Binding Site Ltd., Birmingham, UK) were used. The precipitation ring diameters were measured using Digital Rid Plate Reader. (Binding Site Ltd., Birmingham, UK) The reference range for serum lysozyme was between 9.6 and 16.8 mg/L for all age groups.

### Statistical analysis

Statistical analysis was performed by using SPSS for Windows 13.0.1 (SPSS Inc., Chicago, IL, USA) statistical software products. All results were expressed as mean ± SD. All statistical analyses were performed two-tailed and *p* < 0.05 was considered as significant. One-way ANOVA and Tukey’s tests were used for age distribution, and Chi Square test is used for sex distribution. Multivariate analysis of covariance was used to determine the significance of differences in serum levels of ACE and lysozyme with age as the covariate. Bonnferoni analysis was used to assess pairwise comparisons between the groups.

## Results

### Demographics of the patient and control groups

The age range, mean, standard deviation (SD), and the lower and upper bounds of 95 % confidence interval (CI) values for mean values of age for patients with AS, BD, presumed sarcoidosis, presumed latent TB, presumed latent syphilis, and control group were shown in Table 1. The age range of total 280 subjects was between 9 and 86 with a mean (SD) of 42.892 (16.013). The mean (SD) ages of patients with AS, BD, presumed sarcoidosis, presumed latent TB, presumed latent syphilis and control group were 41.868 (12.536), 38.277 (14.681), 41.000 (18.954), 52.735 (15.525), 44.272 (14.553), and 33.090 (15.377) years respectively (Table 1). The sex distribution and percentages within the groups were shown in Table 2. The total of 280 subjects 139 (49.6 %) were female and 141 (50.4 %) were male. The sex distribution and percentages of the patients with AS, BD, presumed sarcoidosis, presumed latent TB, presumed latent syphilis, and control group were 35 (46.10 %) female and 41 (53.9) male, 43 (59.7 %) female and 29 (40.3 %) male, 13 (41.9 %) female and 18 (58.1 %) male, 36 (52.9 %) female and 32 (47.1 %) male, 4 (36.4 %) female and 7 (63.6 %) male, and 8 (36.4 %) female and 14 (63.6 %) male respectively (Table 2).

**Table 1:** The sample size, age range, mean value, standard deviation, the lower and upper bounds of 95 % confidence interval for mean age of the patients with ocular involvement of ankylosing spondylitis, behcet’s disease, presumed sarcoidosis, presumed latent tuberculosis, presumed latent syphilis and control group

Groups	n	Min age	Max age	Mean age	SD	95 % CI for Mean Lower Bound	95 % CI for Mean Upper Bound
AS	76	15	74	41.868	12.536	37.003	44.733
BD	72	9	75	38.277	14.681	34.827	41.727
P. Sarcoidosis	31	10	72	41.000	18.954	34.047	47.952
TB	68	21	86	52.735	15.525	48.977	56.493
P. Syphilis	11	22	67	44.272	14.553	34.495	54.050
Control	22	12	68	33.090	15.377	26.272	39.908
Total	280	9	86	42.892	16.013	41.009	44.776

*n* Sample size, *Min* minimum, *Max* maximum, *SD* standard deviation, *CI* confidence interval, *AS* ankylosing spondylitis, *BD* behcet’s disease, *TB* presumed latent tuberculosis, *P. Sarcoidosis* presumed sarcoidosis, *P. Syphilis* presumed latent syphilis

**Table 2:** The sex distribution and sample size of patients with ocular involvement of ankylosing spondylitis, behcet’s disease, presumed sarcoidosis and presumed latent tuberculosis, presumed latent syphilis and control group

Groups	Female	Male	n
AS	35 (46.1 %)	41 (53.9 %)	76
BD	43 (59.7 %)	29 (40.3 %)	72
P. Sarcoidosis	13 (41.9 %)	18 (58.1 %)	31
TB	36 (52.9 %)	32 (47.1 %)	68
P. Syphilis	4 (36.4 %)	7 (63.6 %)	11
Control	8 (36.4 %)	14 (63.6 %)	22
Total	139 (49.6)	141 (50.4)	280

*n* sample size, *AS* ankylosing spondylitis, *BD* behcet’s disease, *TB* presumed latent tuberculosis, *P. Sarcoidosis* presumed sarcoidosis, *P. Syphilis* presumed latent syphilis

### Serum ACE and lysozyme activities of the patient and control groups

**Fig. 1:**
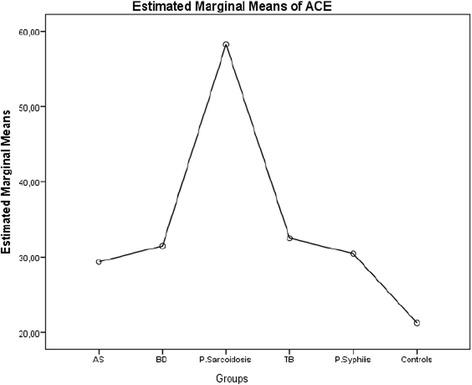
The estimated marginal means of serum angiotensin converting enzyme levels of patients with ocular involvement of ankylosing spondylitis, behcet’s disease, presumed sarcoidosis, presumed latent tuberculosis, presumed latent syphilis and control group

**Fig. 2:**
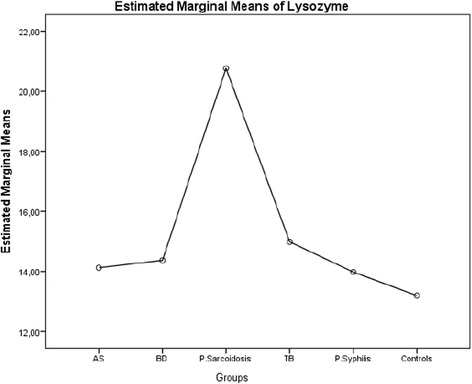
The estimated marginal means of serum lysozyme levels of patients with ocular involvement of ankylosing spondylitis, behcet’s disease, presumed sarcoidosis, presumed latent tuberculosis, presumed latent syphilis and control group

The mean (SD) values of serum ACE and lysozyme levels of the patients with AS, BD, presumed sarcoidosis, presumed latent TB, presumed latent syphilis and control group were shown in Table 3. The mean (SD) values of serum ACE for patients with AS, BD, presumed sarcoidosis, presumed latent TB, presumed latent syphilis and control group were 29.363 (2.012), 31.227 (15.225), 58.164 (35.110), 33.061 (15.065), 30.527 (16.016) and 20.704 (7.962) U/L respectively (Table 3). The estimated marginal means of serum ACE levels in groups were disclosed in Fig. 1. According to this graph, the lowest value for the estimated marginal mean of serum ACE level is 21.251 U/L for control group, and the highest value is 58.274 U/L for the patients with presumed sarcoidosis. The estimated marginal mean values of serum ACE level for the patients with AS, BD, presumed latent TB and presumed latent syphilis were 29.360, 31.392, 32.500 and 30.452 U/L respectively which were lower than the estimated marginal mean value of ACE for presumed sarcoidosis, but higher than the estimated marginal mean value of ACE for control group (Fig. 1). The mean (SD) values of serum lysozyme level for patients with AS, BD, presumed sarcoidosis, presumed latent TB, presumed latent syphilis and control group were 14.096 (4.586), 14.244 (5.358), 20.712 (6.780), 15.259 (8.516), 14.018 (6.679) and 12.927 (4.720) mg/L respectively (Table 3). The estimated marginal means of serum lysozyme levels in groups were disclosed in Fig. 2. According to this graph, the lowest value for the estimated marginal mean of serum lysozyme level was13.200 mg/L for control group, and the highest value was 20.776 mg/L for patients with presumed sarcoidosis. The estimated means of serum lysozyme levels of patients with AS, BD, presumed latent TB and presumed latent syphilis were 14.122, 14.374, 14.998 and 13.989 mg/L respectively which were lower than the estimated marginal mean value of lysozyme for presumed sarcoidosis, and higher than the estimated marginal mean value of serum lysozyme for control group (Fig. 2).

**Table 3:** The sample size, mean value, standard deviation of serum angiotensin converting enzyme levels of the patients with ocular involvement of ankylosing spondylitis, behcet’s disease, presumed sarcoidosis, presumed latent tuberculosis, presumed latent syphilis and control group

Groups	Mean value	SD	95 % CI Lower bound	95 % CI Upper bound
Serum ACE Level (U/L)				
AS	29.363	2.012	25.403	33.324
BD	31.486	2.091	27.369	35.603
P. Sarcoidosis	58.270	3.151	52.067	64.472
TB	32.504	2.254	28.066	36.942
P. Syphilis	30.449	5.285	20.044	40.854
Control	21.254	3.800	13.772	28.736
Serum Lysozyme Level (mg/L)				
AS	14.124	0.718	12.710	15.538
BD	14.371	0.747	12.901	15.841
P. Sarcoidosis	20.765	1.125	18.550	22.979
TB	14.987	0.805	13.402	16.571
P. Syphilis	13.980	1.887	10.265	17.695
Control	13.196	1.357	10.525	15.868

*ACE* angiotensin converting enzyme, *AS* ankylosing spondylitis, *BD* behcet’s disease, *CI* confidence interval, *TB* presumed latent tuberculosis, *P. Sarcoidosis* presumed sarcoidosis, *P. Syphilis* presumed latent syphilis, *SD* standard deviation

### Pairwise comparisons of the mean differences of serum ACE levels between groups

The mean (SD) differences of serum ACE levels between the patients with presumed sarcoidosis and AS, BD, presumed latent TB, presumed latent syphilis and control group were 28.907 (3.735), 26.784 (3.770), 25.776 (3.898), 27.821 (6.155), and 37.016 (4.918) U/L respectively. (Table 4) The increase in serum ACE level was significant for patients with presumed sarcoidosis compared to patients with AS (*p* = 0.0001), BD (*p* = 0.0001), presumed latent TB (*p* = 0.0001), presumed latent syphilis (*p* = 0.0001), and control group (*p* = 0.0001). The mean (SD) differences of serum ACE levels between control group and AS, BD, presumed latent TB, presumed latent syphilis were −8.110 (4.288), −10.232 (4.285), −11.250 (4.528), and −9.195 (6.520) respectively (Table 4). There were no significant differences of serum ACE levels between control group and AS (*p* = 0.895), control group and BD (*p* = 0.264), control group and TB, (*p* = 0.204) control group and presumed latent syphilis. (*p* = 1.000) (Table 4) Pairwise comparison of mean (SD) difference of serum ACE level between AS and BD was −2.123 (2.894), between AS and presumed latent TB was −3.141 (3.038), between AS and presumed latent syphilis was −1.086 (5.656), between BD and presumed latent TB was −1.018 (3.149), between BD and presumed latent syphilis was 1.037 (5.689), and between presumed latent TB and presumed latent syphilis was 2.055 (5.734) respectively (Table 4). No significant differences of serum ACE levels revealed between AS and BD (*p* = 0.689), between AS and presumed latent TB (*p* = 1.000), between AS and presumed latent syphilis (*p* = 0.996), between BD and presumed latent TB (*p* = 1.000), between BD and presumed latent syphilis (*p* = 0.817), and between presumed latent TB and presumed latent syphilis (*p* = 0.504), (Table 4).

**Table 4:** Pairwise comparison of the mean difference values of serum angiotensin converting enzyme levels of the patients with ocular involvement of ankylosing spondylitis, behcet’s disease, presumed sarcoidosis, presumed latent tuberculosis, presumed latent syphilis and control group

Pairwise Comparison	Mean Difference	Standard Deviation	Sig^b^	95 % CI Lower Bound	95 % CI Upper Bound
AS - BD	−2.123	2.894	0.689	−10.691	6.446
AS - P. Sarcoidosis	−28.907	3.735	0.0001	−39.969	−17.845
AS - TB	−3.141	3.038	1.000	−12.137	5.856
AS - P. Syphilis	−1.086	5.656	0.996	−17.836	15.665
AS - Control	8.110	4.288	0.895	−4.590	20.809
BD - AS	2.123	2.894	0.689	−6.446	10.691
BD – P. Sarcoidosis	−26.784	3.770	0.0001	−37.948	−15.620
BD - TB	−1.018	3.149	1.000	−10.343	8.306
BD - P. Syphilis	1.037	5.689	0.817	−15.812	17.885
BD - Control	10.232	4.285	0.264	−2.458	22.922
P. Sarcoidosis - AS	28.907	3.735	0.0001	17.845	39.969
P. Sarcoidosis - BD	26.784	3.770	0.0001	15.620	37.948
P. Sarcoidosis - TB	25.766	3.898	0.0001	14.222	37.310
P. Sarcoidosis–P. Syphilis	27.821	6.155	0.0001	9.593	46.048
P. Sarcoidosis–Control	37.016	4.918	0.0001	22.453	51.580
TB - AS	3.141	3.038	1.000	−5.856	12.137
TB - BD	1.018	3.149	1.000	−8.306	10.343
TB - P. Sarcoidosis	−25.766	3.898	0.0001	−37.310	14.222
TB - P. Syphilis	2.055	5.734	0.504	−14.925	19.095
TB - Control	11.250	4.528	0.204	−2.159	24.660
P. Syphilis - AS	1.086	5.656	0.996	−6.124	5.837
P. Syphilis - BD	−1.037	5.689	0.817	−17.885	15.812
P. Syphilis–P. Sarcoidosis	−27.821	6.155	1.000	−19.035	14.925
P. Syphilis - Control	9.195	6.520	1.000	−10.113	28.504
Control - AS	−8.110	4.288	0.895	−20.809	4.590
Control - BD	−10.232	4.285	0.264	−22.922	2.458
Control –P. Sarcoidosis	−37.016	4.918	0.0001	−51.580	−22.453
Control - TB	−11.250	4.528	0.204	24.660	2.159
Control - P. Syphilis	−9.195	6.520	1.000	−28.504	10.113

The mean difference is significant at the 0.05 level

*Sigb* Adjustment for multiple comparisons Bonferroni, *DV* Dependent variable, *ACE* Angiotensin converting enzyme, *AS* ankylosing spondylitis, *BD* behcet’s disease, *TB* presumed latent tuberculosis, *P. Sarcoidosis* presumed sarcoidosis, *P. Syphilis* presumed latent syphilis

### Pairwise comparisons of the mean differences of serum lysozyme levels between groups

The mean (SD) differences of serum lysozyme levels between patients with presumed sarcoidosis and AS, BD, presumed latent TB, presumed latent syphilis and control group were 6.641 (1.334), 6.394 (1.346), 5.778 (1.392), 6.785 (2.198), and 7.568 (1.756) mg/L respectively (Table 5). The increase in serum lysozyme level was significant for patients with presumed sarcoidosis with respect to AS (*p* = 0.0001), BD (*p* = 0.0001), presumed latent TB (*p* = 0.001), presumed latent syphilis (*p* = 0.033), and control group (*p* = 0.0001) (Table 5) The mean (SD) differences of serum lysozyme levels between control group and AS, BD, presumed latent TB, presumed latent syphilis were −0.928 (1.531), −1.175 (1.530), −1.790 (1.617), and −0.784 (2.328) respectively (Table 5). There were no significant differences of serum lysozyme levels between control group and AS (*p* = 1.000), control group and BD (*p* = 1.000) control group and presumed latent TB (*p* = 1.000), control group and presumed latent syphilis (*p* = 1.000) (Table 5). Pairwise comparison of mean (SD) difference of serum lysozyme level between AS and BD was −0.247 (1.033), between AS and presumed latent TB was −0.863 (1.085), between AS and presumed latent syphilis was 0.144 (2.020), between BD and presumed latent TB was −0.616 (1.124), between BD and presumed latent syphilis was 0.391 (2.031), and between presumed latent TB and presumed latent syphilis was 1.007 (2.047) (Table 5). There were no significant differences of serum lysozyme levels between AS and BD (*p* = 1.000), between AS and presumed latent TB (*p* = 1.000), between AS and presumed latent syphilis (*p* = 1.000), between BD and presumed latent TB (*p* = 1.000), between BD and presumed latent syphilis (*p* = 1.000), and between presumed latent TB and presumed latent syphilis (*p* = 1.000) (Table 5).

**Table 5:** Pairwise comparison of the mean difference values of serum lysozyme levels of the patients with ocular involvement of ankylosing spondylitis, behcet’s disease, presumed sarcoidosis, presumed latent tuberculosis, presumed latent syphilis and control group

Pairwise Comparison	Mean Difference	Standard Deviation	Sig.b	95 % CI Lower Bound	95 % CI Upper Bound
AS - BD	−0.247	1.033	1.000	−3.306	2.812
AS - P. Sarcoidosis	−6.641	1.334	0.0001	−10.590	−2.691
AS - TB	−0.863	1.085	1.000	−4.075	2.349
AS - P. Syphilis	0.144	2.020	1.000	−5.837	6.124
AS - Control	0.928	1.531	1.000	−3.607	5.462
BD - AS	0.247	1.033	1.000	−2.812	3.306
BD – P. Sarcoidosis	−6.394	1.346	0.0001	−10.380	−2.408
BD - TB	−0.616	1.124	1.000	−3.945	2.713
BD - P. Syphilis	0.391	2.031	1.000	−5.625	6.407
BD - Control	1.175	1.530	1.000	−3.356	5.705
P. Sarcoidosis - AS	6.641	1.334	0.0001	2.691	10.590
P. Sarcoidosis - BD	6.394	1.346	0.0001	2.408	10.380
P. Sarcoidosis - TB	5.778	1.392	0.001	1.656	9.900
P. Sarcoidosis–P. Syphilis	6.785	2.198	0.033	0.277	13.293
P. Sarcoidosis – Control	7.568	1.756	0.0001	2.369	12.768
TB - AS	0.863	1.085	1.000	−2.349	4.075
TB - BD	0.616	1.124	1.000	−2.713	3.945
TB - P. Sarcoidosis	−5.778	1.392	0.001	−9.900	−1.656
TB - P. Syphilis	1.007	2.047	1.000	−5.056	7.069
TB - Control	1.790	1.617	1.000	−2.997	6.578
P. Syphilis - AS	−0.144	2.020	1.000	−6.124	5.837
P. Syphilis - BD	−0.391	2.031	1.000	−6.407	5.625
P. Syphilis – P. Sarcoidosis	−6.785	2.198	0.033	−13.293	−0.277
P. Syphilis - TB	−1.007	2.047	1.000	−7.069	5.056
P. Syphilis - Control	0.784	2.328	1.000	−6.110	7.678
Control - AS	−0.928	1.531	1.000	−5.462	3.607
Control - BD	−1.175	1.530	1.000	−5.705	3.356
Control – P. Sarcoidosis	−7.568	1.756	0.0001	−12.768	−2.369
Control - TB	−1.790	1.617	1.000	−6.578	2.997
Control - P. Syphilis	−0.784	2.328	1.000	−7.678	6.110

The mean difference is significant at the 0.05 level

*Sigb* Adjustment for multiple comparisons Bonferroni, *CI* Confidence Interval, *ACE* Angiotensin converting enzyme, *AS* ankylosing spondylitis, *BD* behcet’s disease, *TB* presumed latent tuberculosis, *P. Sarcoidosis* presumed sarcoidosis, *P. Syphilis* presumed latent syphilis

## Discussion

Previous studies have demonstrated the correlations of serum levels of inflammatory chemokines and cytokines with serum ACE and lysozyme activities in patients with ocular and pulmonary sarcoidosis [14, 15]. Chemokine (C-X-C motif) ligand 9 (CXCL9), also known as monokine induced by gamma interferon, (MIG) and C-X-C motif chemokine 10 (CXCL10) also known as interferon gamma-induced protein 10 (IP-10) have been reported to induce the recruitment of activated T helper (Th1) cells [16, 17]. Serum levels of CXCL9 and CXCL10 have been correlated with serum ACE levels in presumed ocular sarcoidosis [15]. Interleukin 12 (IL-12) p40, an essential component of IL-12 has been reported to induce differentiation of naïve Th cells into Th1 cells [15]. Serum concentrations of IL-12 p40 have been found significantly higher in pulmonary sarcoidosis than healthy subjects and have been correlated with serum levels of ACE and lysozyme [18]. This study compares for the first time the serum ACE and lysozyme levels of patients diagnosed with ocular involvement of autoimmune diseases such as AS, BD and presumed sarcoidosis and ocular involvement of infectious diseases such as presumed latent TB and presumed latent syphilis. Intraocular inflammation compatible with sarcoidosis associated with elevated serum ACE levels is considered suggestive for diagnosis of presumptive sarcoidosis in patients without having a tissue biopsy [16, 17, 19–21]. The serum ACE level is disclosed higher in children than in adults [21]. The World Association of Sarcoidosis and Other Granulomatous disorders (WASOG) considered an increased ACE might help in diagnosis of sarcoidosis in children [22]. The age range in sarcoidosis group in our study is between 10 and 72 with a mean age of 41. The estimated mean level of serum ACE of our patients with presumed sarcoidosis is 58.274 IU/L which is above the normal adult range of 8–52 IU/L. Serum ACE level has been compared between patients having sarcoidosis and sarcoidosis-like lung diseases including TB, fibrosing alveolitis, histiocytosis X, and pneumoconiosis [23]. Rise of serum ACE activity has been found in 93 % of patients with active sarcoidosis, 41 % of patients with TB and 56 % of patients with nonspecific inflammatory lung diseases including fibrosing alveolitis, histiocytosis X, and pneumoconiosis [23]. Serum ACE level has not been studied before in the ocular involvement of presumed latent TB. Our study revealed the estimated marginal mean of serum ACE level in patients with presumed latent TB as 32.500 U/L. Pairwise comparison of serum ACE levels between patients with presumed latent TB and presumed sarcoidosis disclosed a statistically significant difference for patients with presumed sarcoidosis with respect to presumed latent TB (*p* = 0.0001). There was also no significant difference of elevated serum ACE levels between patients with presumed latent TB and control group (*p* = 0.204).

The serum activity of ACE has been compared for patients with BD, Vogt-Koyanagi-Harada’s disease and sarcoidosis [24]. Significant elevation of serum ACE activity has been observed only for patients with sarcoidosis [24]. Our study revealed the estimated marginal mean of serum ACE level for patients with BD as 31.392 U/L. A significant elevation of serum ACE level in patients with presumed sarcoidosis with respect to BD has been disclosed in our study (*p* = 0.0001). Serum levels of ACE activity have been compared in patients with rheumatoid arthritis, osteoarthritis, ankylosing spondylitis, psoriatic arthritis and BD, and no statistically significant differences have been found from those of normal controls [25]. The serum ACE activity has not been investigated in HLAB27 positive AS related uveitis. Our study disclosed the estimated marginal mean of serum ACE level in patients with AS as 29.363 U/L. Our study revealed a statistically significant difference in serum ACE level for patients with presumed sarcoidosis compared to patients with AS (*p* = 0.0001). No significant difference of elevated serum ACE levels was found between AS and control group. (*p* = 1.000) Elevated serum lysozyme levels have been included in the criteria for diagnosis of ocular sarcoidosis [26]. The serum levels of ACE and lysozyme have been reported increased in 40 and 42 % of patients with biopsy-proven ocular sarcoidosis respectively [27]. The levels of at least one of the serum markers ACE and lysozyme were found elevated in 58 % of patients with biopsy-proven sarcoidosis [27]. The estimated marginal means of serum ACE and lysozyme levels for the patients with AS, BD, presumed sarcoidosis, presumed latent TB, presumed latent syphilis and control group disclosed on Graph 1 and Graph 2 in our study have similar curves with minimum values for control group and maximum values for presumed sarcoidosis. The sensitivity of serum lysozyme for predicting sarcoidosis was reported as 79.1 % and the sensitivity of serum ACE for predicting sarcoidosis was reported as 59 % [28]. However, the specificity of serum lysozyme level has been reported to be less for sarcoidosis than serum ACE level, and it has been considered that the diagnostic value of serum lysozyme for sarcoidosis might be limited [28]. In another study considering the predictive value of serum ACE and lysozyme levels in diagnosis of ocular sarcoidosis, the sensitivity of increased serum ACE level was found as 84 %, specificity was 95 %, and predictivity was 47 %. However, the sensitivity of increased serum lysozyme levels was 60 %, specificity was 76 %, and spredictivity was 12 % [29]. Both studies showed that increased serum lysozyme levels might be less predictive for ocular sarcoidosis. Our study revealed the estimated marginal means of serum lysozyme for patients with AS, BD, presumed sarcoidosis, presumed latent TB, presumed latent syphilis and control group as 14.124, 14.371, 20.765,14.987, 13.980, and 13.196 mg/L respectively. Pairwise comparisons of the serum lysozyme levels between patients with presumed sarcoidosis and AS and BD revealed statistically significant differences for presumed sarcoidosis (*p* = 0.0001 and *p* = 0.0001 respectively). Pairwise comparison of the serum lysozyme levels between patients with presumed sarcoidosis and presumed latent TB revealed a statistically significant difference for sarcoidosis with the *p* value 0.001 which was greater than the p value of pairwise comparison of the serum ACE levels between patients with presumed sarcoidosis and presumed latent TB. Pairwise comparison of the serum lysozyme levels between the patients with presumed sarcoidosis and presumed latent syphilis revealed a statistically significant difference for presumed sarcoidosis with the *p* value 0.033 which was also greater than the p value of pairwise comparison of the serum ACE levels between patients with presumed sarcoidosis and presumed latent syphilis. Our study disclosed that increase in serum lysozyme levels is more significant than increase in serum ACE levels for patients with infectious uveitis such as presumed latent TB and presumed latent syphilis. This study also revealed that the increase in serum lysozyme levels is less specific than increase in serum ACE levels for presumed sarcoidosis, and elevated serum lysozyme levels might be much more commonly detected in infectious uveitis such as presumed latent TB and presumed latent syphilis than autoimmune uveitis such as AS and BD.

## Conclusions

Elevated serum ACE levels are significant for patients with presumed sarcoidosis compared to ocular involvement of other autoimmune diseases such as BD and AS, and ocular involvement of infectious diseases such as presumed latent TB and presumed latent syphilis. However, elevated serum lysozyme levels might be also detected in ocular involvement of infectious diseases such as presumed latent TB and presumed latent syphilis.

## Abbreviations

ACE: angiotensin converting enzyme
AS: ankylosing spondylitis
BD: behcet’s disease
CI: confidence interval
CSF: cerebrospinal fluid
CXCL: chemokine
D: dilution of the serum. ACE activity is given in units where 1 U is equivalent to the cleavage of 1 μmol of FAPGG in 1 min
FAPGG: N-[3-(2-furyl) acryloyl]-L-phenylalanyl-glycylglycine
FTAABS: fluorescent treponemal antibody absorption
IP: interferon gamma-induced protein
k: change in optical density upon the complete cleavage of 1 μmol of FAPGG
MHA-TP: microhemagglutination assay for Treponema pallidum
RAS: renin angiotensin system
RPR: rapid plasma reagin
S: rate of observed decrease in optical density
SD: standard deviation
Th: T helper
VDRL: venereal disease research laboratory test
WASOG: World Association of Sarcoidosis and Other Granulomatous disorders
